# Role of Intracellular Drug Disposition in the Response of Acute Myeloid Leukemia to Cytarabine and Idarubicin Induction Chemotherapy

**DOI:** 10.3390/cancers15123145

**Published:** 2023-06-11

**Authors:** Gabriela Rodríguez-Macías, Oscar Briz, Candela Cives-Losada, María C. Chillón, Carolina Martínez-Laperche, Ibon Martínez-Arranz, Ismael Buño, Marcos González-Díaz, José L. Díez-Martín, Jose J. G. Marin, Rocio I. R. Macias

**Affiliations:** 1 Experimental Hepatology and Drug Targeting (HEVEPHARM) Group, Biomedical Research Institute of Salamanca (IBSAL), University of Salamanca, 37007 Salamanca, Spain; gabriela.rodriguez@salud.madrid.org (G.R.-M.); obriz@usal.es (O.B.); candelacives@usal.es (C.C.-L.); jjgmarin@usal.es (J.J.G.M.); 2 Department of Hematology, Gregorio Marañón General University Hospital, 28007 Madrid, Spain; cmartinezl@salud.madrid.org (C.M.-L.); ismaelbuno@iisgm.com (I.B.); jdiezm@salud.madrid.org (J.L.D.-M.); 3 Center for the Study of Liver and Gastrointestinal Diseases (CIBERehd), Carlos III National Institute of Health, 28029 Madrid, Spain; 4 Hematology, Biomedical Research Institute of Salamanca, Salamanca University Hospital, 37007 Salamanca, Spain; mcchillon@saludcastillayleon.es (M.C.C.); margondi@usal.es (M.G.-D.); 5 CIBER in Oncology (CIBER-ONC), Carlos III National Institute of Health, 28029 Madrid, Spain; 6 Gregorio Marañón Health Research Institute (IiSGM), 28007 Madrid, Spain; 7 OWL Metabolomics, Bizkaia Technology Park, 48160 Derio, Spain; imartinez@owlmetabolomics.com; 8 Department of Cell Biology, School of Medicine, Complutense University of Madrid, 28040 Madrid, Spain; 9 Department of Medicine, School of Medicine, Complutense University of Madrid, 28040 Madrid, Spain

**Keywords:** AML, blood cancer, chemotherapy, chemoresistance, prognosis

## Abstract

**Simple Summary:**

The impact of genes involved in drug transport and metabolism in regard to the lack of response of acute myeloid leukemia (AML) cells to induction chemotherapy using cytarabine and idarubicin has been investigated in blast cells collected at diagnosis. The aim of this study was to evaluate the usefulness of measuring their expression in order to predict the response to induction chemotherapy. In AML patients with a lower response, the elevated expression of uptake and export transporters and enzymes was found. Additionally, AML cell lines more sensitive to cytarabine showed altered levels of these genes. In conclusion, the poor response of AML patients to chemotherapy can be associated with the increased expression of inactivating enzymes, likely resulting in a reduced intracellular concentration of the active cytarabine metabolite in their blasts.

**Abstract:**

Despite its often low efficacy and high toxicity, the standard treatment for acute myeloid leukemia (AML) is induction chemotherapy with cytarabine and idarubicin. Here, we have investigated the role of transporters and drug-metabolizing enzymes in this poor outcome. The expression levels (RT-qPCR) of potentially responsible genes in blasts collected at diagnosis were related to the subsequent response to two-cycle induction chemotherapy. The high expression of uptake carriers (ENT2), export ATP-binding cassette (ABC) pumps (MDR1), and enzymes (DCK, 5-NT, and CDA) in the blasts was associated with a lower response. Moreover, the sensitivity to cytarabine in AML cell lines was associated with ENT2 expression, whereas the expression of ABC pumps and enzymes was reduced. No ability of any AML cell line to export idarubicin through the ABC pumps, MDR1 and MRP, was found. The exposure of AML cells to cytarabine or idarubicin upregulated the detoxifying enzymes (5-NT and DCK). In AML patients, 5-NT and DCK expression was associated with the lack of response to induction chemotherapy (high sensitivity and specificity). In conclusion, in the blasts of AML patients, the reduction of the intracellular concentration of the active metabolite of cytarabine, mainly due to the increased expression of inactivating enzymes, can determine the response to induction chemotherapy.

## 1. Introduction

One of the main problems in the management of patients with acute myeloid leukemia (AML) is that a significant number of cases indicate a poor response to available chemotherapy, or relapse due to the development of chemoresistance, which leads to very unsatisfactory outcomes [1]. The administration of the antimetabolite arabinosylcytosine (ara-C or cytarabine) as a continuous infusion for 7 days, in combination with an anthracycline, such as idarubicin, given as a daily bolus infusion for the first 3 days (the so-called 7 + 3 regimen), is the standard initial treatment for <65-year-old patients with AML. This treatment reaches complete remission (CR) in about 75% of cases, after one or two cycles. The remaining 25% of patients are classified as primary refractory, or non-responders (NR). This group is characterized by a poor prognosis. Even among responders, relapse occurs in 60–70% of cases within 5 years [2]. Intrinsic and acquired drug resistance following drug exposure are essential events accounting for this poor outcome in AML patients. One of the mechanisms of chemoresistance (MOC) is the reduction of active agents able to reach their intracellular targets in cancer cells. Decreased intracellular concentrations can be due to the impaired expression or function of transporters involved in drug uptake, enhanced drug export through ATP-binding cassette (ABC) pumps, and changes in the expression of enzymes involved in drug metabolism [1,3].

Cytarabine is taken up across the plasma membrane of leukemia cells by members of the equilibrative (ENT) and concentrative (CNT) families of nucleoside transporters [4]. Additionally, cytarabine is exported out of cancer cells by ABC proteins belonging to the family of multidrug resistance-associated proteins (MRP), such as MRP4, MRP5, and MRP8 [5,6]. Once inside the leukemia cells, cytarabine is transformed into its active triphosphate derivative which, upon incorporation into the DNA strand, blocks any subsequent DNA replication [7]. Although three enzymes participate in the formation of the active cytarabine metabolite, deoxycytidine kinase (DCK) catalyzes the rate-limiting step [8]. Moreover, other enzymes reduce the intracellular concentration of the active cytarabine metabolite. This is the case for 5′-nucleotidase (5-NT), which reverses the DCK-mediated reaction, and for cytidine deaminase (CDA), which produces an inactive metabolite [9]. Intracellular levels of the active metabolite of cytarabine (cytarabine triphosphate) have been associated with the ratio of mRNA levels of DCK/5-NT enzymes in primary AML cells [10]. Similarly, cross-resistance between cytarabine and other deoxynucleoside analogs and even between other drugs with different modes of action, such as etoposide and daunorubicin, has been reported in children with acute leukemia [11].

There is some controversy regarding the mechanism accounting for idarubicin (4-demethoxy-daunorubicin) hydrochloride uptake. In aqueous solution at physiological pH, this compound has a net positive charge. However, it has been proposed that due to its marked lipophilicity, idarubicin can cross the plasma membrane by means of a flip-flop mechanism [12]. In contrast, other studies support a role of nucleoside transporters [13] and the organic cation transporter 1 (OCT1), such as is the case of daunorubicin [14] in idarubicin uptake. On the other hand, the contribution of ABC proteins in idarubicin efflux is unclear. Several studies have reported an association between multidrug resistance protein 1 (MDR1) overexpression and the poor outcome of patients treated with idarubicin [15,16], while others described no such association [17,18]. High expression of MRP1 and MRP2, along with breast cancer resistance protein (BCRP or ABCG2), have been proposed as independent predictors of treatment outcome in AML, and the presence of polymorphisms in these genes has been associated with anthracycline response and cardiotoxicity [19,20,21,22,23]. Regarding anthracycline biotransformation, it is mainly performed in non-cancer cells by hepatic enzymes. Thus, the contribution of enzymes expressed in leukemia cells is expected to have a minor impact on pharmacological idarubicin activity [1,24].

Our study aimed to investigate the role of transporters involved in the uptake and efflux of cytarabine and idarubicin and the presence of drug-metabolizing enzymes in leukemia cells (hereafter referred to as blasts) in the response of AML patients to induction chemotherapy and also to evaluate whether their expression levels in blasts collected at diagnosis could be useful to predict the failure of this treatment.

## 2. Materials and Methods

### 2.1. Study Population and Elegibility

Blasts were obtained from the bone marrow samples of 67 patients from the Salamanca University Hospital and the Gregorio Marañón General University Hospital with newly diagnosed AML between 2008 and 2016. Patients were eligible if they received standard front-line induction therapy 7 + 3, consisting of a first cycle of cytarabine (200 mg/m^2^ for 7 days), plus intravenous idarubicin (12 mg/m^2^ from days one to three), and an equal second cycle, or one consisting of a cytarabine and/or idarubicin dose adjustment, if cryopreserved samples at diagnosis were available. The research protocol was approved by the Ethics Committees for Clinical Research of Salamanca University Hospital and Gregorio Marañón University Hospital, and informed written consent was obtained from each patient for the samples to be used for biomedical research.

Clinical and laboratory test values were collected from electronic and paper medical records. The response to induction chemotherapy was assessed according to the revised Cheson criteria [25]. Complete remission (CR) was defined as marrow blasts <5%, transfusion independence, neutrophil count >1 × 10^9^/L, and platelet count >100 × 10^9^/L. When only the first two requirements were met, the status was considered as CR with incomplete recovery (CRi). Patients were classified as responders (R) if they achieved CR or CRi after two cycles. Patients dying during induction and before response assessment were considered as non-evaluable. The remaining patients were classified as non-responders (NR).

### 2.2. Cell Lines

Cell lines MOLM-13, HL-60, HEL, derived from AML, and K-562, derived from chronic myeloid leukemia in the acute phase resembling AML, were purchased from the ATCC or DSMZ (Braunschweig, Germany) cell line repositories and were cultured in RPMI medium supplemented with 10% heat-inactivated fetal calf serum and penicillin-streptomycin and were incubated at 37 °C in a humidified atmosphere with 5% CO_2_. All cell lines were routinely tested by PCR to ensure that they were free of Mycoplasma contamination (Mycoplasma Gel Detection Kit, Biotools, Madrid, Spain).

### 2.3. Cell Viability Assay

Cell viability was evaluated with the MTT-formazan test using thiazolyl blue tetrazolium bromide (MTT) (Sigma-Aldrich, Madrid, Spain). Briefly, the cell suspensions were diluted with media containing graded concentrations of cytarabine or idarubicin, alone or in combination with inhibitors. Next, the cell suspensions were seeded in 96-well microtiter plates (10,000 cells/well, and total volume 150 μL/well). All drug concentrations and controls (untreated cells) were assayed in triplicate in each culture. After 72 h, MTT was added to each well, and the microplates were maintained at 37 °C with 5% CO_2_ for 4 h. Purple formazan crystals were solubilized overnight in 100 µL of 10% SDS in HCl 0.01 M, and subsequently, the absorbance was measured in a microplate reader at 595 nm. The drug concentration required to reduce cell viability by 50% (LC_50_) was calculated from the dose–response curves.

### 2.4. Determination of Gene Expression

Total RNA was extracted from mononuclear cells that were isolated from bone marrow samples using Ficoll-Paque Plus (Amersham Biosciences, Cytiva Europe, Barcelona, Spain), or from cell lines using the illustra RNAspin Mini RNA Isolation Kit (GE Healthcare Life Sciences, Barcelona) [26]. Reverse transcription (RT) was performed using a high-capacity cDNA reverse transcription kit (Applied Biosystems, Thermo Fisher Scientific, Madrid, Spain). Quantitative PCR (QPCR) was performed using AmpliTaq Gold polymerase, and the detection of amplified products was carried out using SYBR™ Green I PCR Master Mix in an ABI Prism 7300 Sequence Detection System, all from Applied Biosystems. The thermal conditions were as follows: 1 cycle of 95 °C for 10 min, followed by 40 cycles of 95 °C for 15 s and 60 °C for 60 s. At the end of each reaction, a melting curve analysis was performed. The primer oligonucleotide sequences to carry out QPCR are described in Table 1. All primer pairs detected the major variant of each gene. The 2^−ΔΔCt^ method was applied to analyze the relative expression of each gene. The results of mRNA abundance for target genes in each sample were normalized compared to GAPDH mRNA abundance. For each cell line, the analysis was performed in cDNA obtained from three independent cell cultures, and the quantification was performed in duplicate. In each human sample, all analyses were carried out in triplicate.

### 2.5. Functional Studies

The functional activity of ABC proteins was assessed by flow cytometry using the fluorescent compounds and specific inhibitors described in Table 2. Briefly, the cells were incubated in 100 μl of the “transport medium” (TM: 96 mM NaCl, 5.3 mM KCl, 1.1 mM KH_2_PO_4_, 0.8 mM MgSO_4_, 1.8 mM CaCl_2_, 11 mM glucose, and 50 mM buffer HEPES/Tris, pH 7.40) containing 50 nM DiOC2(3), 0.1 µM calcein AM, 1 µM carboxyfluorescein, or 25 μM mitoxantrone at 37 °C for 30 min (loading period). Then, the cells were diluted to 1:10 with a substrate-free TM in the presence or absence of a typical inhibitor of each pump, i.e., verapamil (MDR1 inhibitor), probenecid (MRP1-5 inhibitor), diclofenac (MRP3-5 inhibitor), or fumitremorgin C (BCRP inhibitor) (Table 2) and incubated at 37 °C for 30 min. After adding 900 μL of ice-cold TM to stop the transport process, the intracellular fluorescence was immediately determined by flow cytometry.

The contribution of ABC pumps to idarubicin and doxorubicin efflux was also investigated in HEL and MOL-13 cells using flow cytometry. The cells were incubated with 5 µM idarubicin or 25 µM doxorubicin at 37 °C for 30 min (loading period) and then diluted to 1:10 with a substrate-free TM, with or without the selected ABC inhibitors, and incubated at 37 °C for 30 min. After adding 900 μL of ice-cold TM, the fluorescence of these anthracyclines was determined.

### 2.6. Statistical Analysis

Either means and standard error or median and range were used to describe continuous variables. Two-tailed independent Student’s *t*-tests or Kruskal–Wallis H-tests were used to compare groups (paired or unpaired). Categorical variables were subjected to the Chi-square test. Unadjusted *p*-values below 0.05 were regarded as statistically significant. Analysis of the receiver operating characteristic (ROC) curve was completed to evaluate the discrimination ability of the variables. Analysis was performed in terms of area under the receiver-operating characteristic curve (AUC) using R software v4.0.3 (packages: ‘ROCR’ v1.0-11; ‘pROC’ v1.17.0.1). Multivariate analysis, including principal component analysis (PCA), was also performed using this software.

## 3. Results

### 3.1. Characteristics of the Study Population

A total of 75 bone marrow samples were obtained from AML patients at two Spanish hospitals. A total of eight samples were excluded from the study due to insufficient RNA being extracted from the isolated blasts or due to early death that precluded the analysis of the association with the treatment response. The main clinical characteristics of the patients recruited at diagnosis are summarized in Table 3. Gender distribution was balanced in both groups of patients (R and NR). The median age was 53 and 59 years in the R and NR groups, respectively, with a broad age range. Only seven patients were older than 65 years, all of them belonging to the R group. The ranges of platelet count and bone marrow blasts were also very broad in both groups of patients, the median of the latter being higher in R (73%) than in NR (58%) patients. Regarding the cytogenetic risk, only one patient included in the R group showed a favorable risk. Additionally, 65% of R patients and 77% of NR patients belonged to the intermediate group. Interestingly, of the 21 patients with adverse cytogenetic risk, 18 (85.7%) were in the R group, and 3 (14.3%) were in the NR group.

According to the mutational status of nucleophosmin 1 (NPM1) and the fms-related tyrosine kinase 3-internal tandem duplication (FLT3-ITD) of ELN 2017 risk stratification [27], and considering all patients, 55.2% exhibited intermediate risk (NPM1−/FLT3−) and 19.4% showed low risk (NPM1+/FLT3−); the risk was unknown for the group of FLT3+ patients, since the FLT3 allelic ratio was not available for all patients, and the table shows the results of FLT3-positive patients in relation to NPM1 mutational status. The distribution of R and NR patients in the different groups was not markedly different.

The potential clustering of the different groups of patients according to the expression of the selected genes was assessed by multivariate data analysis and unsupervised PCA. No differences were found in gene expression profiles considering the hospital of origin (Supplementary Figure S1A), leucocyte count (Supplementary Figure S1B), percentage of blasts at diagnosis (Supplementary Figure S1C), patients requiring or not requiring allogeneic stem cell transplant (Supplementary Figure S1D), cytogenetic risk (Supplementary Figure S1E), and molecular risk according to NPM1/FLT3-ITD mutational status (Supplementary Figure S1F), since patients clustered together, irrespective of the criteria considered, and a trend regarding group segregation was only detected if the hospital of origin of the samples and the percentage of blasts at diagnosis were considered.

A random distribution of patients that achieved CR or CRi after one cycle (R1) or two cycles (R2) of induction therapy, or with no response after the two cycles of treatment (NR), was found (Supplementary Figure S2). Since the initial analysis of these results showed no differences between R1 and R2, both groups were merged and considered together as responders (R) in further analyses.

### 3.2. Relationship between the Expression of Drug Transporters and Enzymes and the Response to Induction Therapy

**Figure 1:**
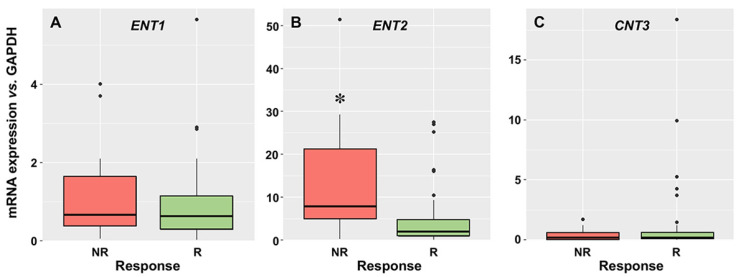
mRNA levels of equilibrative nucleoside transporter 1 (*ENT1*) (**A**), *ENT2* (**B**), and concentrative nucleoside transporter 3 (*CNT3*) (**C**), normalized to *GAPDH*, as determined by RT-QPCR, in the blasts of AML patients at diagnosis who later achieved complete response, even with incomplete recovery (R, *n* = 54) or did not respond (NR, *n* = 13) after two cycles of induction treatment. *, *p* < 0.05.

The mRNA levels of transport proteins involved in cytarabine uptake determined in AML blasts obtained at diagnosis are shown in Figure 1. The order of mRNA abundance was ENT2 > ENT1 ≈ CNT3. Regarding ENT1 and CNT3 mRNA, no differences in levels between R and NR patients were found. Surprisingly, ENT2 expression was significantly higher in NR than in R patients. CNT1 and CNT2 mRNA was undetectable in the blasts.

**Figure 2:**
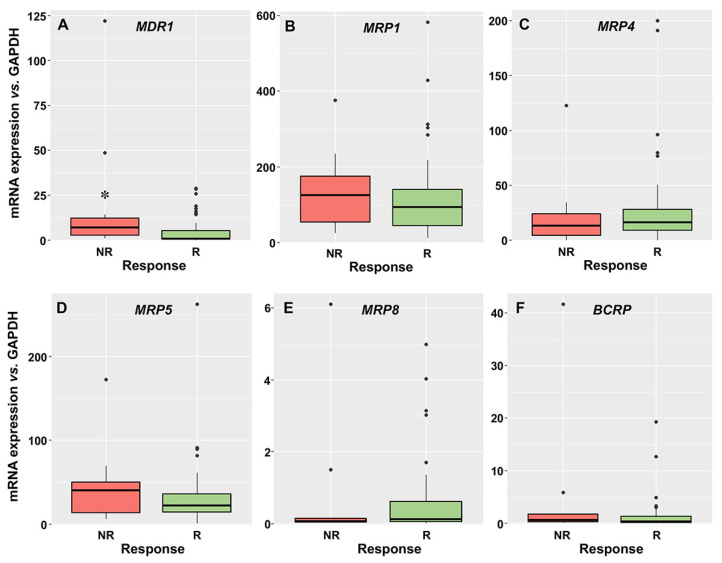
mRNA levels of multidrug resistance 1 (*MDR1*) (**A**), multidrug resistance-associated protein 1 (*MRP1*) (**B**), *MRP4* (**C**), *MRP5* (**D**), *MRP8* (**E**) and breast cancer resistance protein (*BCRP*) (**F**), normalized to GAPDH, as determined by RT-QPCR, in blasts of AML patients at diagnosis who later achieved complete response, even with incomplete recovery (R, *n* = 54), or did not respond (NR, *n* = 13) after two cycles of induction treatment. *, *p* < 0.05.

Regarding export pumps, the order of mRNA abundance was MRP1 > MRP5 > MRP4 > MDR1 > BCRP > MRP8. Higher levels of MDR1 were found in NR than in R patients (Figure 2A), and no significant difference was found between groups for MRP1, MRP4, MRP5, MRP8, and BCRP mRNA abundance (Figure 2B–F).

**Figure 3:**
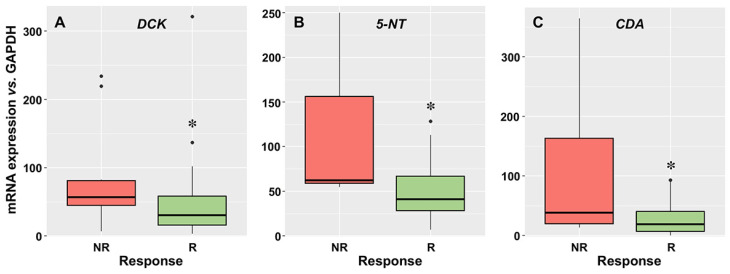
mRNA levels of deoxycytidine kinase (*DCK*) (**A**), 5′-nucleotidase (*5-NT*) (**B**), and cytidine deaminase (*CDA*) (**C**), normalized to GAPDH, as determined by RT-QPCR, in blasts of AML patients at diagnosis who later achieved complete response, even with incomplete recovery (R, *n* = 54), or did not respond (NR, *n* = 13) after two cycles of induction treatment. *, *p* < 0.05.

The expression of DCK was lower in the blasts of R patients (Figure 3A), which seems contradictory, since considering the role of DCK cytarabine activation, its downregulation would be consistent with a lower intracellular proportion of the active compounds. However, this could be compensated by the fact that there was a significantly higher expression of the two enzymes responsible for cytarabine inactivation, i.e., 5-NT (Figure 3B) and CDA (Figure 3C), in the blasts of NR patients.

We investigated whether there was any association between the expression of the genes of interest. Supplementary Table S1 shows the correlation between each pair of genes. A low grade of collinearity was observed, since values ≥0.75 were only found for DCK and 5-NT.

**Figure 4:**
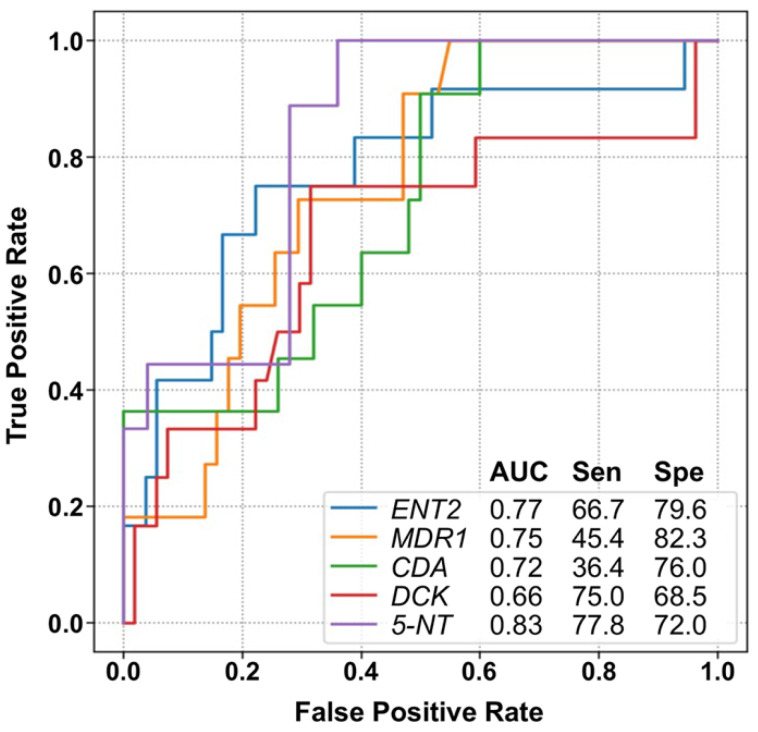
Area under the receiver operating characteristic curve (AUC), sensitivity (Sen), and specificity (Spe) of the five genes identified in the blasts of AML patients at diagnosis which were selected to predict the response in AML patients to induction chemotherapy. 5-NT, 5′-nucleotidase; CDA, cytidine deaminase; DCK, deoxycytidine kinase; ENT2, equilibrative nucleoside transporter 2; MDR1, multidrug resistance protein.

High levels of ENT2, MDR1, DCK, 5-NT, and CDA mRNA in the blasts at diagnosis were associated with a lower response to induction therapy. Figure 4 shows the AUC, sensitivity, and specificity values for each independent gene.

Two genes, namely 5-NT and DCK, showed higher values of sensitivity and acceptable specificity, permitting the correct detection of ≥75% of the AML patients who will respond to induction chemotherapy.

We then tested whether the combination of two or more of these genes associated with chemoresistance increased the ability to predict the lack of response to treatment. The analysis revealed that when considering the upregulation of two of these genes, the sensitivity and specificity results were 58.3% and 85.2%, respectively. If enhanced expression of three genes was considered as the selection criterion, the values showed 50% sensitivity and 96.3% specificity. Finally, if the upregulation of four genes was used for the analysis, the specificity was optimal (100%), but the sensitivity dropped to 16.7%.

### 3.3. Sensitivity of Leukemic Cells to Cytarabine and Idarubicin

The cytostatic effects of cytarabine and idarubicin were determined in a panel of three AML human cell lines; HL-60, MOLM-13, and HEL, as well as one derived from chronic myeloid leukemia in the acute phase, resembling AML (K-562).

The dose–response curves of cytarabine and idarubicin are shown in Supplementary Figures S3 and S4, respectively. Table 4 shows the comparison of the concentrations inducing lethal effect in 50% of these cells (LC_50_). The sensitivity to cytarabine was higher for HL-60 > HEL ≈ MOLM-13 >> K-562, and the sensitivity to idarubicin was higher for MOLM-13 > HL-60 > HEL >> K-562.

### 3.4. Effect of Drug Exposure of Transporters and Enzymes in AML Cell Lines

**Figure 5:**
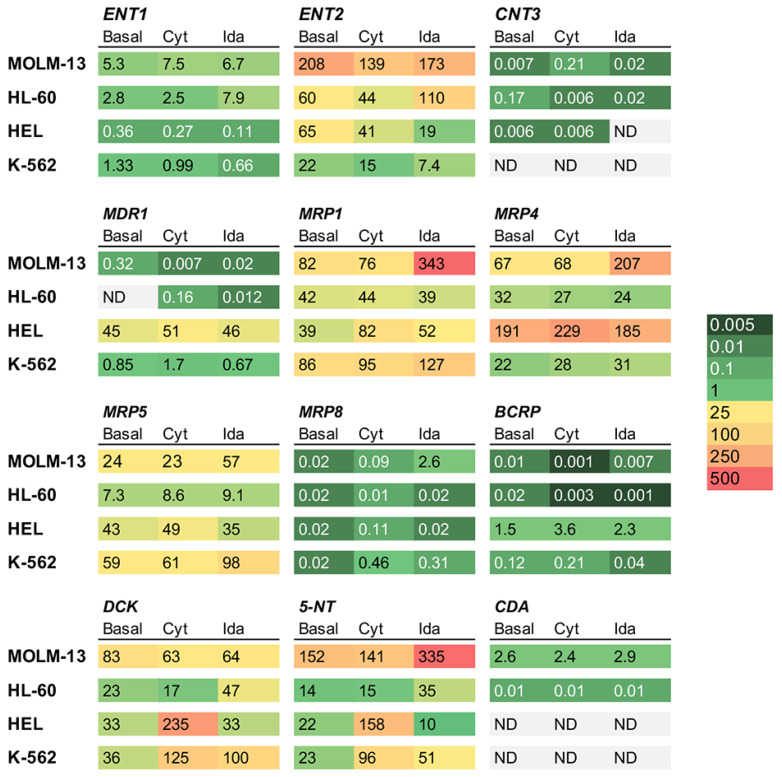
Basal and cytarabine- or idarubicin-induced mRNA expression, normalized to *GAPDH*, of transporters involved in the uptake and efflux of cytarabine (Cyt) and idarubicin (Ida), as well as the enzymes involved in cytarabine metabolism in MOLM-13, HL-60, HEL, and K-562 cells, as determined by RT-QPCR. The values are the means of three different cultures, determined in duplicate (SD < 10%). Cells were incubated for 72 h in the absence (basal) or presence of cytarabine or idarubicin using the LC_50_ concentration of each drug in each cell line. ND, not detected; 5-NT, 5′-nucleotidase; BCRP, breast cancer resistant protein; CDA, cytidine deaminase; CNT, concentrative nucleoside transporter; DCK, deoxycytidine kinase; ENT, equilibrative nucleoside transporter; MDR, multidrug resistance protein; MRP, multidrug resistance-associated protein.

The basal mRNA levels of nucleoside equilibrative transporters ENT1-2 and concentrative transporters CNT1-3 were determined (Figure 5). The CNT1 and CNT2 levels were undetectable in all cell lines, and the *CNT3* levels were low in HL-60 > MOLM-13 ≈ HEL cells and undetectable in K-562 cells. The expression of ENT1 was higher in MOLM-13 > HL-60 > K-562 > HEL. The expression of ENT2 was high in all the cell lines, especially in MOLM-13 > HL-60 ≈ HEL > K-562.

Regarding the basal mRNA levels of export pumps (Figure 5), MDR1 was undetectable in HL-60 cells and highly expressed in HEL >> K-562 > MOLM-13 cells. The expression of BCRP was low in all the cell lines (HEL >> K-562 > HL-60 ≈ MOLM-13). MRP1 mRNA levels were high in all cell lines, especially in MOLM-13 ≈ K-562 > HL-60 ≈ HEL; MRP4 mRNA levels were high in HEL > MOLM-13 > HL-60 > K-562; MRP5 levels occurred in the order of K-562 > HEL> MOLM-13 > HL-60. The expression of MRP8 was very similar, and was low in all cell lines.

Regarding the basal expression of cytarabine metabolism enzymes (Figure 5), the expression of DCK mRNA was higher in MOLM-13 than in the rest of cell lines; however, this cell line also presented a markedly higher expression of 5-NT and CDA involved in cytarabine inactivation.

The exposure to drugs induced changes in the expression of genes associated with chemoresistance (Figure 5). Incubation with cytarabine induced an increased expression of DCK and 5-NT in HEL and K-562 cells and a decreased expression of ENT2 in all the cell lines. Incubation with idarubicin resulted in reduced ENT2 expression in HEL and K-562 and increased MRP1, MRP4, MRP5, and 5-NT expression in MOLM-13 and K-562.

### 3.5. Functional Studies in AML Cell Lines

As previously described [26], the functionality of MDR1 and BCRP in AML-derived cell lines was associated with their expression levels, MDR1 activity was only observed in HEL cells, and very low activity of BCRP was found in all the cell lines.

**Figure 6:**
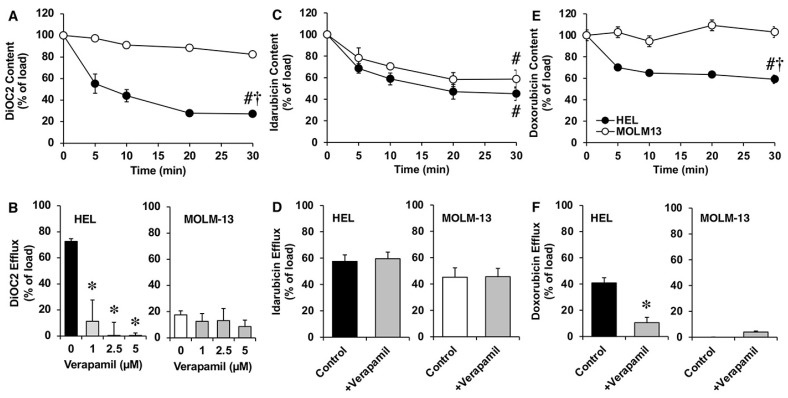
Time course of cell content of the fluorescent MDR1 substrate DiOC2 (**A**), idarubicin (**C**), and doxorubicin (**E**) from preloaded myeloid leukemia cells HEL y MOLM13. Efflux of DiOC2 (**B**), idarubicin (**D**), and doxorubicin (**F**) from preloaded HEL y MOLM13 cells in the presence or absence of verapamil (increasing concentrations in (**B**) and 5 µM in (**D**,**F**)). After the cells were loaded with 50 nM 3,3′-diethyloxacarbocyanine iodide (DiOC2), 5 μM idarubicin, or 25 μM doxorubicin at 37 °C for 30 min (loading period), they were diluted to 1:10 with a substrate-free medium, containing or not containing verapamil, and incubated at 37 °C for 30 min. Values (means ± SEM) were determined by flow cytometry from 3 different cultures. #, *p* < 0.05, as compared to content after the loading period (t = 0). †, *p* < 0.05, as compared with MOLM-13 cells. *, *p* < 0.05, as compared to the substrate content in the absence of verapamil.

Given that there is controversy regarding the ability of MDR1 to transport idarubicin, we selected HEL and MOLM-13 cells, with high and very low levels of this efflux pump, respectively, to compare their ability to export this drug. As shown in Figure 6A, the content of MDR1 substrate DiOC2 decreased progressively for 30 min in HEL cells, but not in MOLM-13 cells. In addition, DiOC2 efflux in HEL cells was inhibited in the presence of increasing concentrations of the MDR1 inhibitor verapamil (Figure 6B).

In both in HEL and MOLM-13 cells, after a loading period with idarubicin, the cell content decreased progressively over 30 min after removing the drug from the incubation medium (Figure 6C). Drug efflux was not affected by the presence of verapamil (Figure 6D). For comparison, doxorubicin (a typical MDR1 substrate) efflux was analyzed. Doxorubicin cell content was reduced over 30 min in HEL, but not in MOLM-13 cells (Figure 6E). Moreover, the presence of verapamil inhibited doxorubicin efflux in HEL cells (Figure 6F).

**Figure 7:**
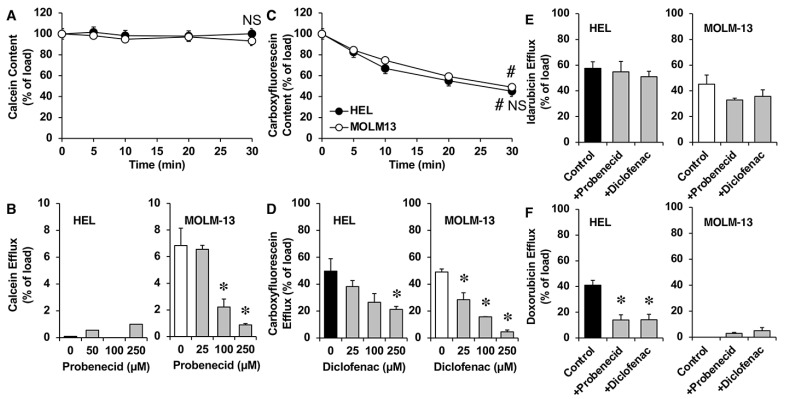
Time course of cell content of the fluorescent MRP1-2 substrate calcein (**A**) or the fluorescent MRP3-5 substrate carboxyfluorescein (**C**) from preloaded HEL and MOLM13 myeloid leukemia cells. Efflux of calcein (**B**), carboxyfluorescein (**D**), idarubicin (**E**), and doxorubicin (**F**) from preloaded HEL and MOLM13 cells in the presence or absence of probenecid (increasing concentrations in (**B**) and 250 µM in (**E**,**F**)) or diclofenac (increasing concentrations in (**D**) and 250 µM in (**E**) and (**F**)). After the cells were loaded with 0.1 μM calcein, 1 μM carboxyfluorescein, 5 μM idarubicin, or 25 μM doxorubicin at 37 °C for 30 min (loading period), they were diluted to 1:10 with a substrate-free medium, containing or not containing inhibitors, and incubated at 37 °C for 30 min. Values (means ± SEM) were determined by flow cytometry from 3 different cultures. #, *p* < 0.05, as compared to content after the loading period (t = 0). NS, *p* > 0.05, as compared with MOLM-13 cells. *, *p* < 0.05, as compared to the substrate content in the absence of inhibitors.

We also investigated the activity of MRPs involved in the efflux of idarubicin. As shown in Figure 7A, calcein (a known MRP1 substrate) efflux was not detectable in HEL cells and was low, although concentration-dependently inhibitable by probenecid (a known MRP1 inhibitor) in MOLM-13 cells (Figure 7B). Cell fluorescence content due to carboxyfluorescein, commonly used in the assessment of MRP4 and MRP5 function, was reduced in both HEL and MOLM-13 cells (Figure 7C). MRP4/MTP5-mediated efflux was reduced in the presence of diclofenac (Figure 7D), demonstrating that MRP4 and MRP5 are functional. Idarubicin efflux was slightly reduced in the presence of probenecid or diclofenac in MOLM-13 cells, but not in HEL cells (Figure 7E), while the doxorubicin efflux was significantly reduced in the presence of both inhibitors in HEL, but not in MOLM-13 cells (Figure 7F).

**Figure 8:**
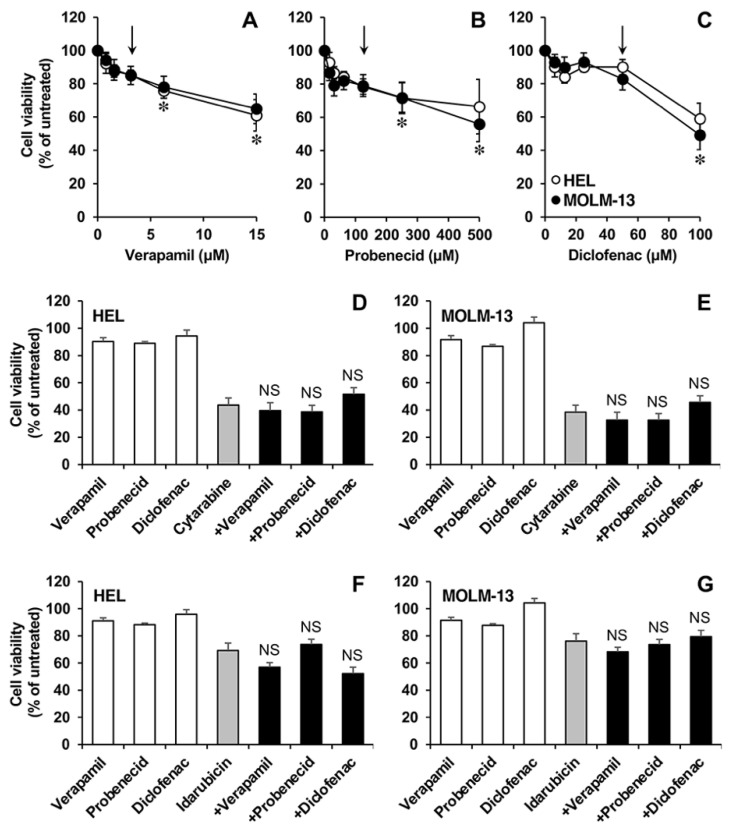
Effect of the combination of cytarabine or idarubicin with ABC protein inhibitors on the viability of the HEL and MOLM-13 cell lines. Cells were incubated in the presence of increasing concentrations of verapamil (a MDR1 inhibitor) (**A**), probenecid (a MRPs inhibitor) (**B**), or diclofenac (an MRPs inhibitor) (**C**) for 72 h to determine subtoxic concentrations of the inhibitors (arrows). HEL and MOLM-13 cells were incubated with 50 nM cytarabine (**D**,**E**) or idarubicin 5 nM (**F**) and 1 nM (**G**), alone or in combination with the chosen concentrations of the inhibitors for 72 h. Cell viability was determined by the MTT-formazan test. Values are means ± SEM of at least 3 experiments performed in triplicate. *, *p* < 0.05, compared to untreated cells. NS, *p* > 0.05, compared with cytarabine or idarubicin alone.

Finally, we investigated whether ABC pump inhibitors can sensitize AML cells to cytarabine and idarubicin as an indirect method of determining whether these transporters are involved in the efflux of these drugs. HEL and MOLM-13 cells were exposed to increasing concentrations of the MDR1 inhibitor verapamil (Figure 8A), the MRP1-5 inhibitor probenecid (Figure 8B), or the MRP3-5 inhibitor diclofenac (Figure 8C) to determine subtoxic concentrations of each compound, i.e., 3.1 µM verapamil, 125 µM probenecid, and 50 µM diclofenac. The cell viability of HEL (Figure 8D) and MOLM-13 (Figure 8E) cells after exposure to 50 nM cytarabine alone for 3 days was the same as that noted in the presence of the selected concentrations of verapamil, probenecid, or diclofenac. Similarly, the cell viability of HEL (Figure 8F) and MOLM-13 (Figure 8G) cells after exposure to 5 nM and 1 nM idarubicin alone for 72 h, respectively, was unaffected by the presence of the selected concentrations of the ABC pump inhibitors verapamil, probenecid, or diclofenac.

## 4. Discussion

The identification of prognostic biomarkers in AML patients is required to help clinicians to choose between conventional and investigational therapies [27] and to adapt the treatment of patients during follow-up, based on their response.

Among the mechanisms responsible for resistance to the drugs administered in induction therapy for AML are changes in the expression of some proteins responsible for the uptake and efflux of cytarabine and idarubicin, as well as the enzymes that activate and inactivate these drugs, as previously described [28,29]. Time course analysis of gene expression during patient treatment represents a feasible approach to identify relevant mechanisms affected by standard induction chemotherapy in patients with AML [30]. In this work, we have analyzed the relationship between gene expression in AML patient blasts and the response to two cycles of induction chemotherapy. Although the results are interesting, it should be noted that a limitation of this study is the small number of NR patients included in this cohort.

The processing of the drugs in the liver may affect their pharmacokinetics and hence, their efficacy and toxicity, but the expression levels of these genes in tumor cells can also determine the amount of active intracellular agents and can play an important role in intrinsic and acquired resistance [1]. In this regard, we have previously demonstrated that the decreased expression of plasma membrane transporters involved in sorafenib uptake, such as OCT1, affects the response of leukemic cells to this drug [26]. It has been proposed that a reduced ENT1 expression, together with the upregulation of inactivating enzymes of cytarabine, especially CDA, can predict a worse treatment outcome in AML, irrespective of cytogenetic and molecular risk groups [4]. In the present study, ENT1 expression was similar in both R and NR AML patients. However, although it seems contradictory, higher ENT2 levels were associated with poor response to treatment. These findings are consistent with an association between ENT2 upregulation and advanced stages of several types of cancers, such as mantle cell lymphoma, hepatocellular carcinoma, and colorectal cancer [31]. Our results suggest that high ENT2 expression is not related to enhanced resistance to cytarabine due to changes in drug transport, but rather it can be a paraphenomenon occurring in tumor cells, likely associated with phenotypic characteristics of higher malignancy, since this transporter facilitates the uptake of purine and pyrimidine nucleosides and nucleobases, which are required to generate new nucleotides for a very active DNA synthesis.

Although there is evidence supporting that MRP4 and MRP5 can transport cytarabine [5,6], no relationship between the response to induction therapy and the levels of these export pumps was found in AML patients. In addition, our in vitro studies showed that the inhibitor diclofenac was not able to sensitize AML cells to cytarabine, calling into question the role of these pumps in the efflux of this drug in regards to the blasts. The low MRP8 expression in these cells suggests a minor role of this transporter in the response to cytarabine.

There is some controversy regarding the role of MDR1 in AML chemoresistance. Although various studies have failed to detect any association between MDR1 expression and the response of AML patients to treatment with induction therapy [17,18], other reports have indicated that high MDR1 expression can predict the lack of response in these patients [19,32,33]. Our results support this concept. In naive AML patients with high MDR1 expression, idarubicin has been associated with better remission induction than daunorubicin [34], which seems contradictory with the generally accepted idea that MDR1 can transport both anthracyclines. However, the ability of ABC pumps to transport idarubicin has not been clearly elucidated. Several studies have found an association between MDR1 overexpression and poor outcome after idarubicin treatment [16], while others describe no such relationship [17,18]. Our results in cells expressing MDR1 suggested that this pump can export doxorubicin, but not idarubicin, supporting the hypothesis that MDR1 upregulation in blasts from NR patients is involved in the mechanisms of chemoresistance developed by AML cells to overcome response to treatment.

Results in AML cell lines showed that changes in transporters can only partially explain drug resistance in these cellular models. HL-60 cells were the most sensitive to cytarabine, which could be associated with high ENT2 expression, as well as low levels of efflux pumps (MRP4 and MRP5) and inactivating enzymes (5-NT and CDA). MOLM-13 cells were the most sensitive to idarubicin, but MRP1 levels were similar to those found in K-562 cells, which are the most resistant to this drug. Nevertheless, low ENT2 expression and high levels of MRP1 and MRP5 alone cannot justify the high resistance to both drugs. Additionally, MDR1 and MRPs inhibitors did not sensitize AML cells to idarubicin or cytarabine.

The balance between the activity of the three enzymes involved in nucleoside metabolism assayed here is one of the crucial factors determining the concentration of the active metabolite of cytarabine within AML cells [8,9]. The loss of DCK in AML cells has been shown to increase the resistance of these cells to cytarabine [35]. In addition, it has been suggested that increased CDA expression and/or activity contributes to decreased plasma cytarabine levels in AML patients and likely contributes to worse outcomes after treatment [36]. Although increased expression of DCK, 5-NT, and CDA was found in NR patients, the changes were more marked for drug inactivating enzymes. It should be noted that other mechanisms of drug resistance may contribute to the lack of response to these drugs; for instance, the high expression of the enzyme SAMHD1, which can hydrolyze and inactivate triphosphorylated nucleoside analogues, has been associated with reduced sensitivity to cytarabine [37] and changes in the levels of the target drug (topoisomerase IIα), in the case of idarubicin [38].

## 5. Conclusions

In blasts from AML patients, the intracellular concentration of the active metabolite of cytarabine is expected to be reduced by the upregulation of the inactivating enzymes 5-NT and CDA. Moreover, the high expression of drug transporters (ENT2 and MDR1) and metabolizing enzymes (DCK, 5-NT, and CDA) in these cells is associated with a worse response to induction therapy. Larger scale validation studies are needed to confirm the clinical diagnostic value of these biomarkers.

## Figures and Tables

**Table 1:** Gene-specific oligonucleotide sequences of primers used in real-time RT-QPCR.

Protein/Gen	Primer Forward (5′-3′)	Primer Reverse (5′-3′)	Acccess Number
ENT1 (*SLC29A1*)	CCATCGATCTGGAGCCCGT	TGTCATGGTGATGGTGTTCTCGGT	NM_001078177
ENT2 (*SLC29A2*)	CCGCCATCCCGTACTTCCA	TGTTGAAGTTGAAGGCATCCTCGG	NM_001532
CNT1 (*SLC28A1*)	GCTCTGCACTGGGCTCTCT	TTGAGAAACCTCCTCAGCTTTGGC	NM_004213
CNT2 (*SLC28A2*)	GGGCTGGAGCTCATGGAAAAAGAA	CCACCGACTCCTCCTCTGGTAA	NM_004212.3
CNT3 (*SLC28A3*)	GGTTCTGGCTGAAGTGGGTGAT	ACATTATGAGCCCACCGAAGGACA	NM_022127
MDR1 (*ABCB1*)	GCGCGAGGTCGGAATGGAT	CCATGGATGATGGCAGCCAAAGTT	NM_000927
BCRP (*ABCG2*)	CCCAGGCCTCTATAGCTCAGATCATT	CACGGCTGAAACACTGCTGAAACA	NM_004827
MRP1 (*ABCC1*)	CCGCTCTGGGACTGGAATGT	GTGTCATCTGAATGTAGCCTCGGT	NM_004996
MRP4 (*ABCC4*)	TGCAAGGGTTCTGGGATAAAGA	CTTTGGCACTTTCCTCAATTAACG	NM_005845
MRP5 (*ABCC5*)	GTTCAGGAGAACTCGACCGTTGG	TTTGGAAGTAGTCCGGATGGGCTT	NM_005688
MRP8 (*ABCC8*)	CGGTCTCCTTTATTCTCCCACA	AGCCTCTTAAACTGGCTGATGAAGT	NM_032583
CDA (*CDA*)	GCTATCGCCAGTGACATGCAAGA	AGTTGGTGCCAAACTCTCTCATGACT	NM_001785
DCK (*DCK*)	GGGAACATCGCTGCAGGGAA	ACAGGTTCAGGAACCACTTCCCA	NM_000788
5-NT (*NT5C2*)	AGAAGCCTATCATCGGGTGTTTGTGAA	CATACTCTGGGGACTTGTACACAGCAA	NM_012229
GAPDH (*GAPDH*)	TGAGCCCGCAGCCTCC	TACGACCAAATCCGTTGACTCC	NM_002046

5-NT, 5′-nucleotidase; BCRP, breast cancer resistant protein; CDA, cytidine deaminase; CNT, concentrative nucleoside transporter; DCK, deoxycytidine kinase; ENT, equilibrative nucleoside transporter; GAPDH, glyceraldehyde-3-phosphate dehydrogenase; MDR, multidrug resistance protein; MRP, multidrug resistance-associated protein.

**Table 2:** Fluorescent substrates and inhibitors used in flow cytometry.

Pumps	Fluorescent Substrates	Inhibitors
MDR1	3,3′-Diethyloxacarbocyanine iodide (DiOC2(3))	Verapamil
MRP1-2	Calcein	Probenecid
MRP3-5	Carboxyfluorescein	Probenecid, Diclofenac
BCRP	Mitoxantrone	Fumitremorgin C (FTC)

BCRP, breast cancer resistance protein; MDR1, multidrug resistance 1; MRP, multidrug resistance-associated protein. Calcein and carboxyfluorescein were added to the transport medium as non-fluorescent esters.

**Table 3:** Characteristics of responder and non-responder patients prior to treatment.

Variable	Responders (*n* = 54)	Non-Responders (*n* = 13)
Age, yr		
Median (Range)	53 (27–75)	59 (44–63)
Female gender, *n* (%)	28 (51.8)	7 (53.8)
WBC count per µL		
Median (Range)	25,100 (900–234,900)	43,800 (2000–141,000)
Platelet count per µL		
Median (Range)	67,500 (6000–321,000)	74,000 (25,000–244,000)
Bone marrow blasts, %		
Median (Range)	73 (20–95)	58 (22–96)
Cytogenetic risk		
Favorable, *n* (%)	1 (1.9)	0 (0)
Intermediate, *n* (%)	35 (64.8)	10 (76.9)
Adverse, *n* (%)	18 (33.3)	3 (23.1)
Molecular risk		
NPM1+/FLT3-ITD−, *n* (%)	10 (18.5)	3 (23.1)
NPM1+/FLT3-ITD+, *n* (%)	8 (14.8)	3 (23.1)
NPM1−/FLT3-ITD−, *n* (%)	31 (57.4)	6 (46.1)
NPM1−/FLT3-ITD+, *n* (%)	5 (9.3)	1 (7.7)

FLT3-ITD, fms-related tyrosine kinase 3-internal tandem duplication; NPM1, nucleophosmin 1; WBC, white blood cells.

**Table 4:** Comparison of LC_50_ in cell lines.

	Cytarabine (nM)	Idarubicin (nM)
MOLM-13	68.3± 4.2	3.5 ± 0.7
HL-60	24.4 ± 1.1	7.5 ± 0.8
HEL	60.2 ± 4.6	17.2 ± 1.0
K-562	5700 ± 624	162 ± 21

Values (means ± SEM) are expressed as percentages of controls (cells incubated in the absence of drugs) from at least three experiments, performed in triplicate.

## Data Availability

Data available on request due to ethical restrictions.

## Supplementary Materials

The following supporting information can be downloaded at: https://www.mdpi.com/article/10.3390/cancers15123145/s1, Figure S1. Principal component analysis (PCA) score plots of selected 12 genes expression in Ficoll-concentrated human blast samples of patients with AML. Colors represent the origin of the samples (A), leukocyte count (B), percentage of blasts at diagnosis (C), patients receiving transplant (D), cytogenetic risk (1: favorable; 2: intermediate; 3: adverse) (E), and molecular risk according to NPM1/FLT3-ITD mutational status (1: low; 2: intermediate; 3: unknown) (F). Each dot represents one sample; Figure S2. Principal component analysis (PCA) score plots of selected 12 genes expression in Ficoll-concentrated human blast samples of patients with AML. Colors represent the response to induction therapy; complete response even with incomplete recovery after one cycle (R1) of induction therapy with cytarabine and idarubicin 7 + 3, after two cycles of treatment (R2), or no response after two cycles of treatment (NR); Figure S3. Effect of cytarabine on viability of MOLM-13 (A), HL-60 (B), HEL (C) and K-562 (D) cell lines. Cells were incubated with increasing concentrations of the drug for 72 h and cell viability was determined by the MTT test. Values are means ± SEM of 3 independent experiments performed in triplicate. *, *p* < 0.05, compared with non-treated cells by Student’s *t*-test; Figure S4. Effect of idarubicin on viability of MOLM-13 (A), HL-60 (B), HEL (C) and K-562 (D) cell lines. Cells were incubated with increasing concentrations of the drug for 72 h and cell viability was determined by the MTT test. Values are means ± SEM of 3 independent experiments performed in triplicate. *, *p* < 0.05, compared with non-treated cells by Student’s *t*-test; Table S1. Collinearity exploratory analysis. Correlations over 0.75 or under -0.75 are highlighted in red.
