# Supplementary material for: Role of Intracellular Drug Disposition in the Response of Acute Myeloid Leukemia to Cytarabine and Idarubicin Induction Chemotherapy
Source: Cancers (Basel). 2023 Jun 11;15(12):3145. doi: 10.3390/cancers15123145 (PMC10296567; doi:10.3390/cancers15123145)
Supplement: Supplementary file 1 [file cancers-15-03145-s001.zip › cancers-2399139-supplementary.pdf]

cancers

MDPI

Article

# Role of Intracellular Drug Disposition in the Response of Acute Myeloid Leukemia to Cytarabine and Idarubicin Induction Chemotherapy

**Gabriela Rodríguez-Macías [1,2], Oscar Briz [1,3], Candela Cives-Losada [1], María C. Chillón [4,5], Carolina Martínez-Laperche [2,6], Ibon Martínez-Arranz [7], Ismael Buño [2,6,8], Marcos González-Díaz [4,5], José L. Díez-Martín [2,6,9], Jose J. G. Marin [1,3] and Rocio I. R. Macias [1,3,*]**

1 Experimental Hepatology and Drug Targeting (HEVEPHARM) Group, Biomedical Research Institute of Salamanca (IBSAL), University of Salamanca, 37007 Salamanca, Spain; gabriela.rodriguez@salud.madrid.org (G.R.-M.); obriz@usal.es (O.B.); candelacives@usal.es (C.C.-L.); jjgmarin@usal.es (J.J.G.M.)
2 Department of Hematology, Gregorio Marañón General University Hospital, 28007 Madrid, Spain; cmartinezl@salud.madrid.org (C.M.-L.); ismaelbuno@iisgm.com (I.B.); jdiezm@salud.madrid.org (J.L.D.-M.)
3 Center for the Study of Liver and Gastrointestinal Diseases (CIBERehd), Carlos III National Institute of Health, 28029 Madrid, Spain
4 Hematology, Biomedical Research Institute of Salamanca, Salamanca University Hospital, 37007 Salamanca, Spain; mcchillon@saludcastillayleon.es (M.C.C.); margondi@usal.es (M.G.-D.)
5 CIBER in Oncology (CIBER-ONC), Carlos III National Institute of Health, 28029 Madrid, Spain
6 Gregorio Marañón Health Research Institute (IiSGM), 28007 Madrid, Spain
7 OWL Metabolomics, Bizkaia Technology Park, 48160 Derio, Spain; imartinez@owlmetabolomics.com
8 Department of Cell Biology, School of Medicine, Complutense University of Madrid, 28040 Madrid, Spain
9 Department of Medicine, School of Medicine, Complutense University of Madrid, 28040 Madrid, Spain
* Correspondence: rociorm@usal.es; Tel.: +34-666-5966-21

## Supplementary information

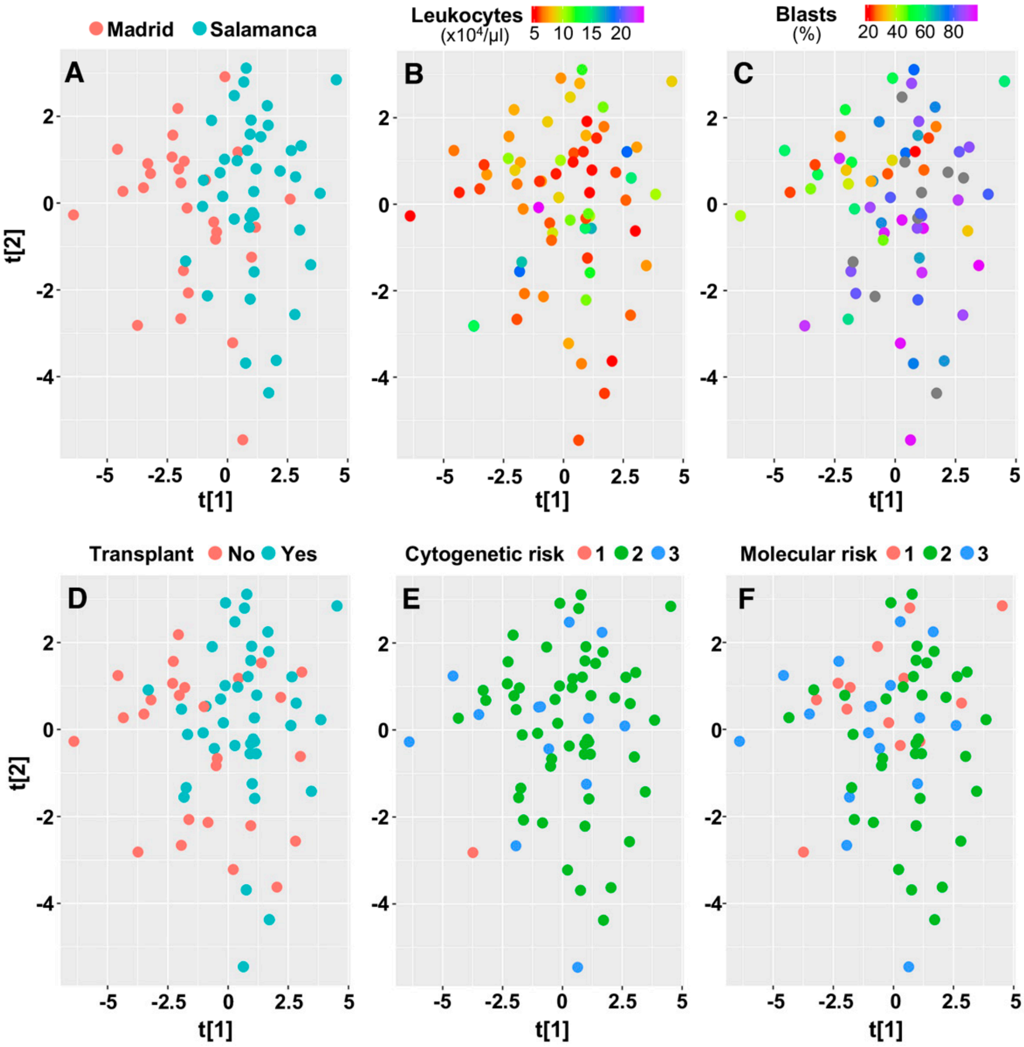


**Supplementary Figure S1.** Principal component analysis (PCA) score plots of selected 12 genes expression in Ficoll-concentrated human blast samples of patients with AML. Colors represent the origin of the samples (**A**), leukocyte count (**B**), percentage of blasts at diagnosis (**C**), patients receiving transplant (**D**), cytogenetic risk (1: favorable; 2: intermediate; 3: adverse) (**E**), and molecular risk according to NPM1/FLT3-ITD mutational status (1: low; 2: intermediate; 3: unknown) (**F**). Each dot represents one sample.

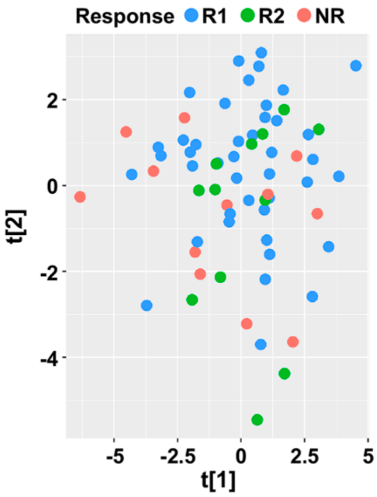


**Supplementary Figure S2.** Principal component analysis (PCA) score plots of selected 12 genes expression in Ficoll-concentrated human blast samples of patients with AML. Colors represent the response to induction therapy; complete response even with incomplete recovery after one cycle (R1) of induction therapy with cytarabine and idarubicin 7+3, after two cycles of treatment (R2), or no response after two cycles of treatment (NR).

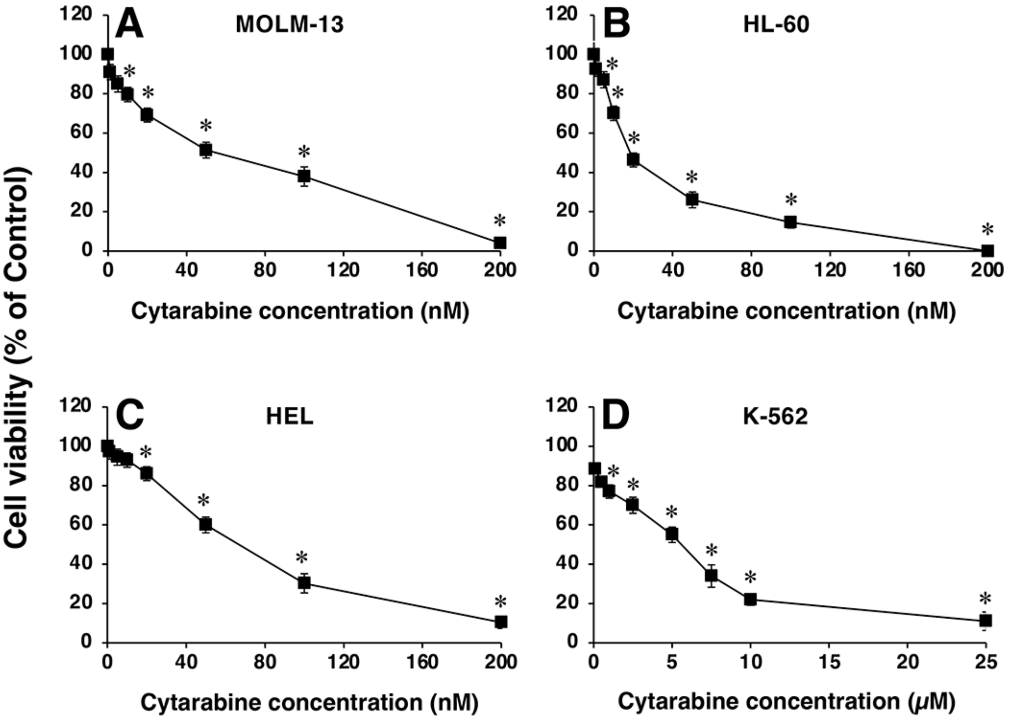


**Supplementary Figure S3.** Effect of cytarabine on viability of MOLM-13 (**A**), HL-60 (**B**), HEL (**C**) and K-562 (**D**) cell lines. Cells were incubated with increasing concentrations of the drug for 72 h and cell viability was determined by the MTT test. Values are means ± SEM of 3 independent experiments performed in triplicate. *, $p < 0.05$, compared with non-treated cells by Student's *t*-test.

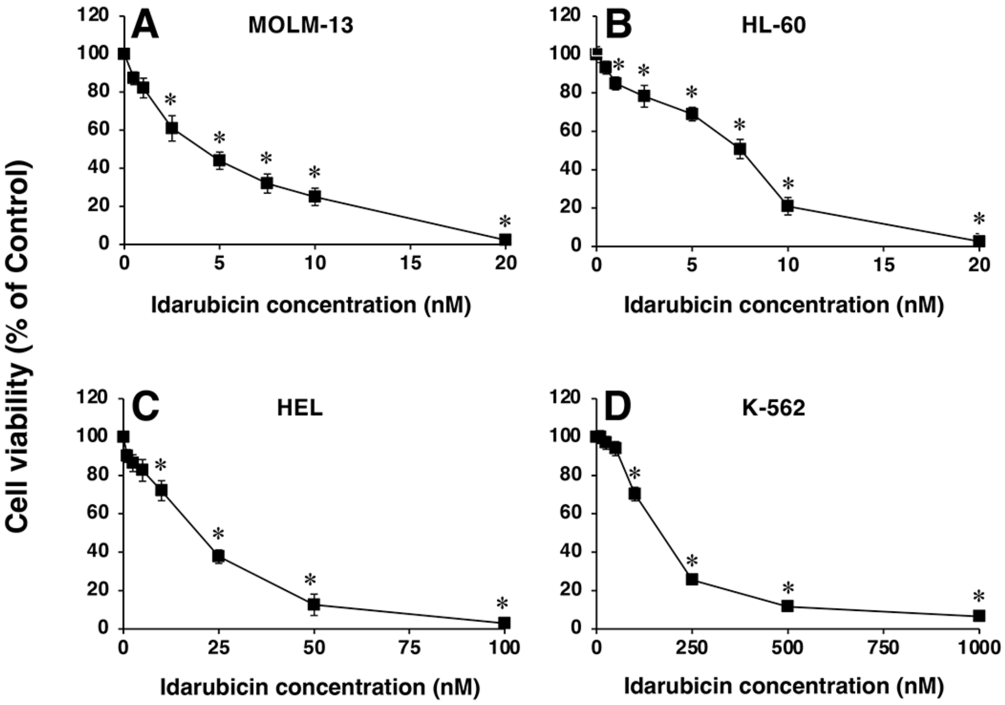


**Supplementary Figure S4.** Effect of idarubicin on viability of MOLM-13 (**A**), HL-60 (**B**), HEL (**C**) and K-562 (**D**) cell lines. Cells were incubated with increasing concentrations of the drug for 72 h and cell viability was determined by the MTT test. Values are means ± SEM of 3 independent experiments performed in triplicate. *, $p < 0.05$, compared with non-treated cells by Student's *t*-test.

**Supplementary Table S1.** Collinearity exploratory analysis. Correlations over 0.75 or under −0.75 are highlighted in red.

| | ENT1 | ENT2 | CNT3 | MDR1 | BCRP | MRP1 | MRP4 | MRP5 | MRP8 | DCK | 5-NT | CDA |
|---|---|---|---|---|---|---|---|---|---|---|---|---|
| **ENT1** | | | | | | | | | | | | |
| **ENT2** | 0.30 | | | | | | | | | | | |
| **CNT3** | -0.05 | -0.16 | | | | | | | | | | |
| **MDR1** | 0.09 | 0.14 | -0.10 | | | | | | | | | |
| **BCRP** | 0.30 | 0.20 | -0.05 | 0.14 | | | | | | | | |
| **MRP1** | 0.20 | 0.59 | -0.20 | 0.21 | 0.24 | | | | | | | |
| **MRP4** | -0.05 | 0.22 | -0.07 | -0.04 | -0.14 | 0.64 | | | | | | |
| **MRP5** | 0.22 | 0.17 | -0.06 | -0.03 | 0.08 | 0.28 | 0.15 | | | | | |
| **MRP8** | 0.17 | 0.02 | -0.06 | -0.13 | 0.02 | -016 | -0.05 | 0.14 | | | | |
| **DCK** | 0.22 | 0.46 | -0.14 | 0.14 | 0.43 | 0.66 | 0.36 | 0.52 | 0.05 | | | |
| **5-NT** | 0.27 | 0.52 | -0.13 | 0.04 | 0.32 | 0.55 | 0.25 | 0.69 | 0.04 | **0.80** | | |
| **CDA** | 0.27 | 0.23 | -0.05 | 0.002 | 0.50 | 0.11 | -0.20 | 0.28 | 0.05 | 0.45 | 0.51 | |

5-NT, 5′-nucleotidase; BCRP, breast cancer resistant protein; CDA, cytidine deaminase; CNT, concentrative nucleoside transporter; DCK, deoxycytidine kinase; ENT, equilibrative nucleoside transporter; MDR, multidrug resistance protein; MRP, multidrug resistance-associated protein.
